# An innovative therapeutic educational program to support older drivers with cognitive disorders: Description of a randomized controlled trial study protocol

**DOI:** 10.3389/fneur.2022.901100

**Published:** 2022-07-18

**Authors:** Floriane Delphin-Combe, Marie-Hélène Coste, Romain Bachelet, Mélissa Llorens, Claire Gentil, Marion Giroux, Laurence Paire-Ficout, Maud Ranchet, Pierre Krolak-Salmon

**Affiliations:** ^1^Memory Clinical and Research Center of Lyon (CMRR), Lyon Institute for Elderly, Hospices Civils de Lyon, Lyon, France; ^2^TS2-LESCOT, Univ Gustave Eiffel, IFSTTAR, Univ Lyon, Lyon, France; ^3^INSERM U1028, CNRS UMR5292, Lyon Neuroscience Research Center, Brain Dynamics and Cognition Team, Lyon, France

**Keywords:** cognitive impairment, driving cessation, education, self-regulation, caregiver

## Abstract

Older drivers face the prospect of having to adjust their driving habits because of health problems, which can include neurocognitive disorders. Self-awareness of driving difficulties and the interaction between individual with neurocognitive disorders and natural caregiver seem to be important levers for the implementation of adaptation strategies and for the subsequent voluntary cessation of driving when the cognitive disorders become too severe. This study aims to evaluate an educational program for patient/natural caregiver dyads who wish to implement self-regulation strategies in driving activity, and to improve self-awareness of driving ability. The ACCOMPAGNE program is based on seven group workshops, which target the dyad. The workshops deal with the impact of cognitive, sensory and iatrogenic disorders on driving. They tackle questions about responsibility, and about autonomy and social life. They also provide alternative solutions aimed at maintaining outward-looking activities even if driving is reduced or stopped. A randomized controlled trial is planned to evaluate the effectiveness of the program 2 months and 6 months after inclusion, and to compare this to the effectiveness of conventional approaches. The main outcome of this trial (i.e., the implementation of self-regulated driving strategies), will be measured based on scores on the “Current Self-Regulatory Practices” subscale of the Driver Perception and Practices Questionnaire. The Driving Habits Questionnaire will be used to measure secondary outcomes (indicators of driving changes; indicators of changes in mood, quality of life and caregiver burden; and self-awareness of driving abilities). Indicators will be collected for both patients and natural caregivers. This cognitive, social and psychological program should allow older individuals with cognitive disorders to drive more safely, and help to maintain the quality of life and mood of both patient and natural caregiver despite driving limitations. The patient's care path would be optimized, as he/she would become an actor in the process of giving up driving, which will, most certainly, be needed at some point in the progress of neurocognitive disorders. This process ranges from becoming aware of driving difficulties, to implementing self-regulation strategies, through to complete cessation of driving when necessary.

**Clinical trial registration number:**

NCT04493957.

## Introduction

Older drivers are faced with the decision to continue or discontinue driving because of health problems, which may also include cognitive disorders. Due to population aging, the number of individuals with major cognitive disorders is increasing and should reach 65.7 million by 2030 (1). Consequently, the number of older drivers with cognitive disorders will increase over the next few years. Individuals with cognitive disorders have an increased risk of traffic accidents (2–4). The risk of individuals with major cognitive disorders being involved in a collision is up to 4.5 times higher than for older people without cognitive disorders (4, 5). Most on-road studies (4, 6–10) and simulator studies (9, 11) have also shown that, on average, the ability to drive is more affected in drivers with cognitive disorders than in drivers without cognitive disorders. Studies which take a naturalistic approach have also shown that drivers with cognitive disorders have poorer self-regulatory behavior than healthy older drivers (12). However, more than 40% of people with cognitive disorders, whose accident risk is 2–5 times higher than older adults without cognitive disorders, continue to drive (13). Almost half of the patients studied were involved in a crash in the 3 years leading up to the diagnosis of cognitive disorder (14). Driving a car may therefore become an important road-safety issue for patients with cognitive disorders.

Giving up driving is a challenging transition for older drivers, and can sometimes be difficult (15). It is a significant life-event, and can lead to major changes in lifestyle, such as a decrease in outward-looking activities, increased loneliness and social isolation. It may also result in depression (16). In France, only a certified physician (i.e., certified by the Prefect of the Department) is qualified to authorize or prohibit driving activity. Medical confidentiality regulations mean that attending physicians, geriatricians and neurologists cannot oblige patients to see their certified physician. They can only advise patients to adapt their driving or give up driving completely. Only patients themselves can impart information about their medical conditions. It is up to the patient or the family caregiver to make an appointment with their certified physician. The patient might then be required to undergo a medical check-up. Then, the certified physician will provide a medical decision about the ability to drive based on the advice of professionals. He/she also specifies the duration of this authorization or prohibition whether any restriction is recommended (e.g., vehicle adaptation, automatic gearboxes). This could lead to suspension of the driving license by the authorities (i.e., the Prefect of the Department). Unfortunately, very few patients take the step as observed in our clinical practice.

A number of intervention programs aimed at managing driving cessation in older adults have been proposed in different countries. A recent review underlined the encouraging results of these intervention programs on processing the decision (17). Only two controlled randomized studies were identified in the course of this review of the literature. One proposed an intervention based on emotional management strategies related to the issue of giving up driving. The results showed a decrease in depressive symptoms in participants who participated in the emotional management intervention, compared to those who did not (control group) (18). The other study proposed an intervention based on an interactive psychoeducational and motivational method for caregivers of patients with cognitive disorders who were still driving. Caregivers in the group which participated in the psychoeducational intervention felt better prepared to discuss giving up driving with the patient, and were less anxious about triggering anger or hurting the patient (19). However, the authors highlighted the lack of methodological consistency in the various studies. A more recent study investigated the effectiveness of a program composed of classroom workshops. These provided peripheral visual field and dynamic vision training, and a driving simulator training session to enable better prediction of driving risk. Results showed a significant increase in safe driving performance in older adults (20). An Australian study is currently being carried out in order to determine the effectiveness of an individualized self-awareness and adjustment program on improving or maintaining mobility after the transition from driving, to driving cessation (21). However, some aspects of driving cessation have never been considered in a cessation management program in older adults with major cognitive disorders. These include: the ability to implement adaptation strategies; the crucial role of the natural caregiver in the cessation process; and work on individuals' self-awareness of their difficulties, aimed at making them actors in the decision to stop driving.

A recent study suggested that older drivers with minor cognitive disorders were more likely to self-regulate their driving than drivers without cognitive disorders (22). These results are consistent with those of Raedt and Ponjaert-Kristoffersen, which showed that adaptation strategies such as avoidance of certain situations (e.g., night-time trips, peak-hour trips, on unfamiliar roads, or on roads with rough or damaged surfaces) may reduce accident risk in older adults with no major cognitive disorders (23). Charlton et al. also confirms that older adults engaged in self-regulatory driving strategies like reducing driving exposure (driving distance and/or frequency) (24). However, only 1 in 5 elderly drivers whose driving performance is declining over time correctly detect this change (25), which may hinder implementation of regulatory strategies. In addition to these factors, it also seems important to improve the use of regulatory strategies that help older drivers to anticipate and prepare for the consequences of driving cessation before it occurs. The implementation of regulatory strategies is modulated by different variables: self-awareness of health issues, the reasons that push people to continue driving (social representation, maintenance of lifestyle habits, independence), and available resources (social and family environment, infrastructure, and legislation) (26). Intervention programs based on these factors showed effectiveness in driving cessation. Studies have been carried out on older people with ophthalmological conditions- but no cognitive disorders - to find out how an educational program affects their perception of the driving difficulties they experience and of any self-regulatory strategies they may use (27). The Driving Habits Questionnaire (DHQ) was used to measure the perception of driving difficulties, and the Driver Perception and Practices Questionnaire (DDPQ) was used to evaluate drivers' attitude to road safety and self-regulation strategies. Six months after the program, results showed improvements in these measurements compared to those recorded before the intervention (e.g., drivers made fewer trips, traveled shorter distances, and avoided visually difficult situations, such as driving at night, or in foggy conditions).

The studies mentioned focus on programs which target either the patient or the caregiver. However, the interaction between patient and natural caregiver appears to be at the heart of this process (17, 28). A recent study showed that spouses play a significant role in their partners' decision to self-regulate their driving (29). The authors pointed out that intervention programs for driving cessation needed to consider the importance of interdependency in couples and its impact on their driving decisions and outcomes.

Finally, work on drivers' self-awareness and on their ability to anticipate their own difficulties also needs to be done (15). Older adults who stop driving appear to be those who are the most aware of their difficulties (26, 30).

The ACCOMPAGNE educational program presented here (ACCompanying Older drivers in the decision to Maintain or abandon the Pursuit of driving Activity in Geriatric and Neurological units) puts precisely this notion of self-awareness at the heart of the program. The objective of the proposed intervention is to help participants to become aware of their driving difficulties. The intervention was designed following the principles of therapeutic patient education. Therapeutic patient education is “a patient-centered process that addresses patient needs, resources, values, and strategies. It allows patients to improve their knowledge and skills in relation to their illness and its treatment” (31). It has positive impacts on the patient quality of life, treatment adherence and reduction in complications in different diseases such as asthma (32), diabetes (33), osteoarthritis (34) or Alzheimer's disease (35).

Natural caregivers play a major role in this intervention, whose aim is to support both the patient and the caregiver in dealing with the psycho-socio-economic consequences of driving restrictions. Indeed, programs combining caregivers and patients interventions has shown to be effective in increasing the general mental health of both caregivers and patients as well as delay the admittance in long-stay care (36). The role of the natural caregiver in the program is also to help the patient to recall the information learned during the program. The majority of patients with cognitive disorders have episodic memory impairment, which makes it difficult for them to memorize new information. This could be an obstacle to the implementation of self-regulation strategies.

### Aims and hypotheses

A randomized, controlled, single-blind trial will be conducted to assess the impact of the ACCOMPAGNE program on the implementation of self-regulatory strategies in the short and long-term in participants with mild to major cognitive disorders. We hypothesize that participants who benefit from the ACCOMPAGNE program will implement more self-regulatory strategies than those who receive conventional recommendations. The implementation of self-regulatory strategies will be assessed 2 and 6 months after the intervention. Because the objective is to investigate the effects of the ACCOMPAGNE program on the implementation of driving strategies, and the link with awareness of driving difficulties, rather than to examine participants' real driving ability, the driving simulator was chosen over on-road testing. This places drivers in a reproducible and controlled driving environment, and will be used to collect objectives as well as subjective measures of driving ability and self-awareness of this ability. For the same reasons, we will use the differences between the points of view of participants and their natural caregivers to measure the changes after the program.

The secondary objectives are to determine the effects of the ACCOMPAGNE program on (a) self-awareness of driving ability, (b) the mood and quality of life of both participant and natural caregiver, and (c) the natural caregiver's burden. We expect that the ACCOMPAGNE program will (a) increase self-awareness of driving ability (b) maintain participants' mood and quality of life despite any changes made, and (c) maintain the mood and quality of life of natural caregivers, and alleviate their burden to a greater degree than conventional care can.

## Methods/design

### Design

A national randomized, controlled, trial will be conducted. Two-hundred dyads (consisting of participants and natural caregivers) will be randomly assigned either to the ACCOMPAGNE group or to a control group. The trial will take place in four centers in France: memory clinics and geriatric units of the University Hospitals of Lyon, Reims and Tours, and the Geriatric Hospital of Mont d'Or. The effects of the ACCOMPAGNE program will be assessed 2 months and 6 months after the intervention. For 40 participants in the center in Lyon, a test will also be conducted on a driving simulator.

### Participants

Inclusion criteria: Participants must be aged between 50 and 95 years-old, must have a current driving license, and have to drive at least twice a week. Participants must have been diagnosed with a major or minor cognitive disorder in accordance with the DSM-V criteria (e.g., Alzheimer's disease, vascular disease). They must score over 18 on the Mini Mental State Examination (MMSE), and be sufficiently able to speak and write French to perform clinical evaluations and participate in workshops. The cut-off value for the MMSE was fixed at 18 because, below 18/30, patients generally present important cognitive disorders which may greatly hinder their involvement, contribution and understanding of the group intervention. Indeed, some authors used the cut-off of 18/30 to discriminate between mild and moderate or severe neurocognitive disorders (37, 38). There is no upper limit since participants are only included if they have a diagnosis of Mild or Major Neurocognitive Disorders. Thus, the presence of cognitive impairment is objectified by other means than the MMSE score.

A family member will have to accompany them and be present for at least 4 h a week. The natural caregivers must be involved in helping the participant with the activities of daily living and be able to speak and write sufficiently well to perform the clinical assessments. All participants will provide free and informed consent.

Exclusion criteria: Participants must not have any history of major psychiatric disorder, alcoholism, they should not be undergoing non-stabilized antidepressant treatment (i.e., it should not have been changed or started <6 months prior to the study), or have any sensory problems which would prevent them from participating in workshops. They must not suffer from any pathology which compromises their health in the short or medium term, and they must be able to express their consent. The natural caregivers must not have any sensory disturbances which would prevent them from participating in workshops.

**Figure 1:**
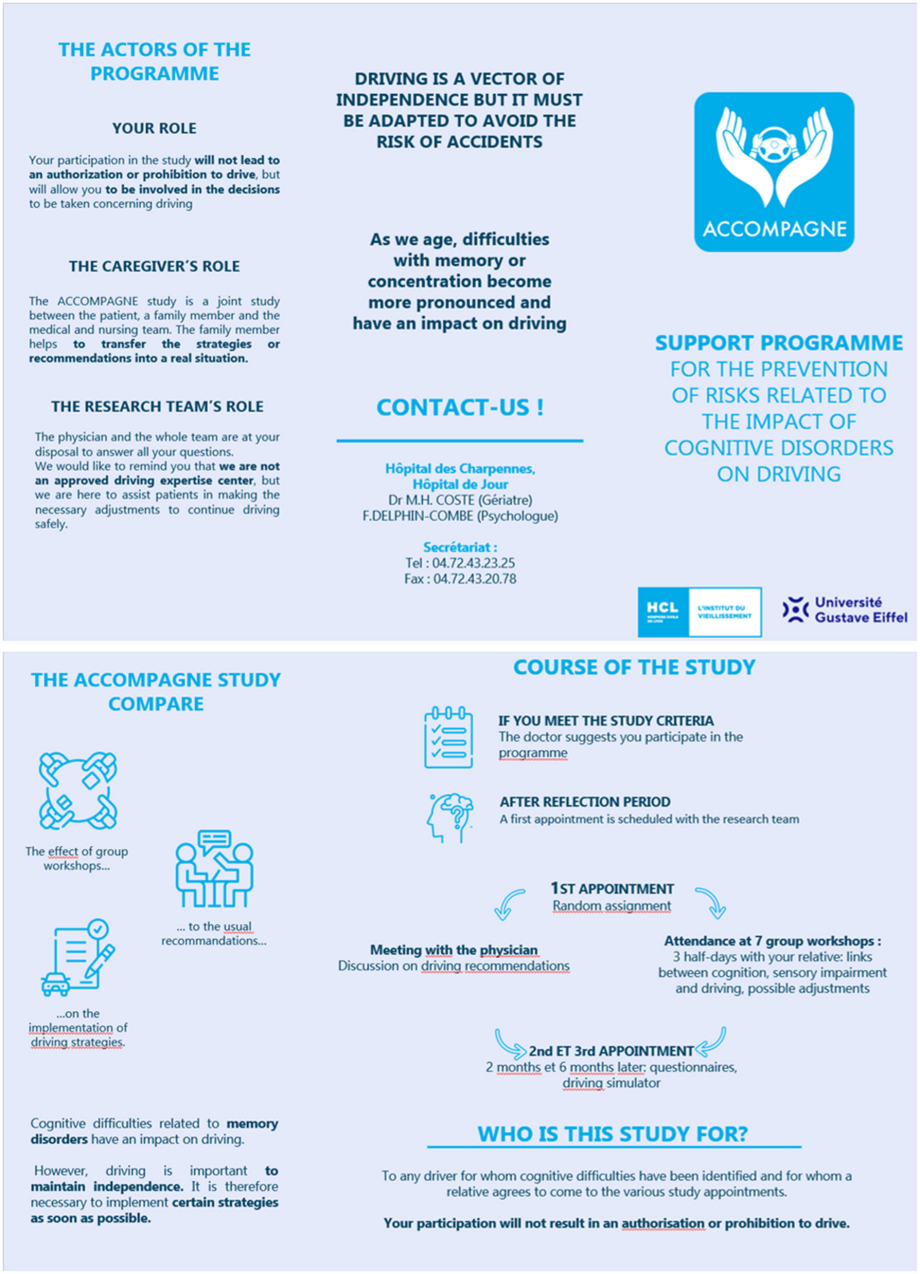
English version of the recruitment leaflet.

To recruit the patient-caregiver dyads corresponding to the inclusion/exclusion criteria, physicians will give them information about the study when seeing them in their care pathway about cognitive complaints. Oral and (comprehensive) written information will be given to them as well as a leaflet summarizing the most important pieces of information about the aims of the study, their roles and what they could expect from it. The fact that the study cannot leads to the authorization or the prohibition of driving was emphasized. You can find the English version of the leaflet in Figure 1.

### Randomization

Randomization between the two groups will be stratified in the individual recruitment centers, based on participants' level of cognitive disorder (two levels: minor or major). Randomization by block permutation will be carried out in order to balance the two groups (experimental and control groups), and to allow the workshops to start in the experimental group, which requires a minimum of four dyads. The block permutations will differ in size to ensure the unpredictability of the random allocation.

### Participant timeline

**Figure 2:**
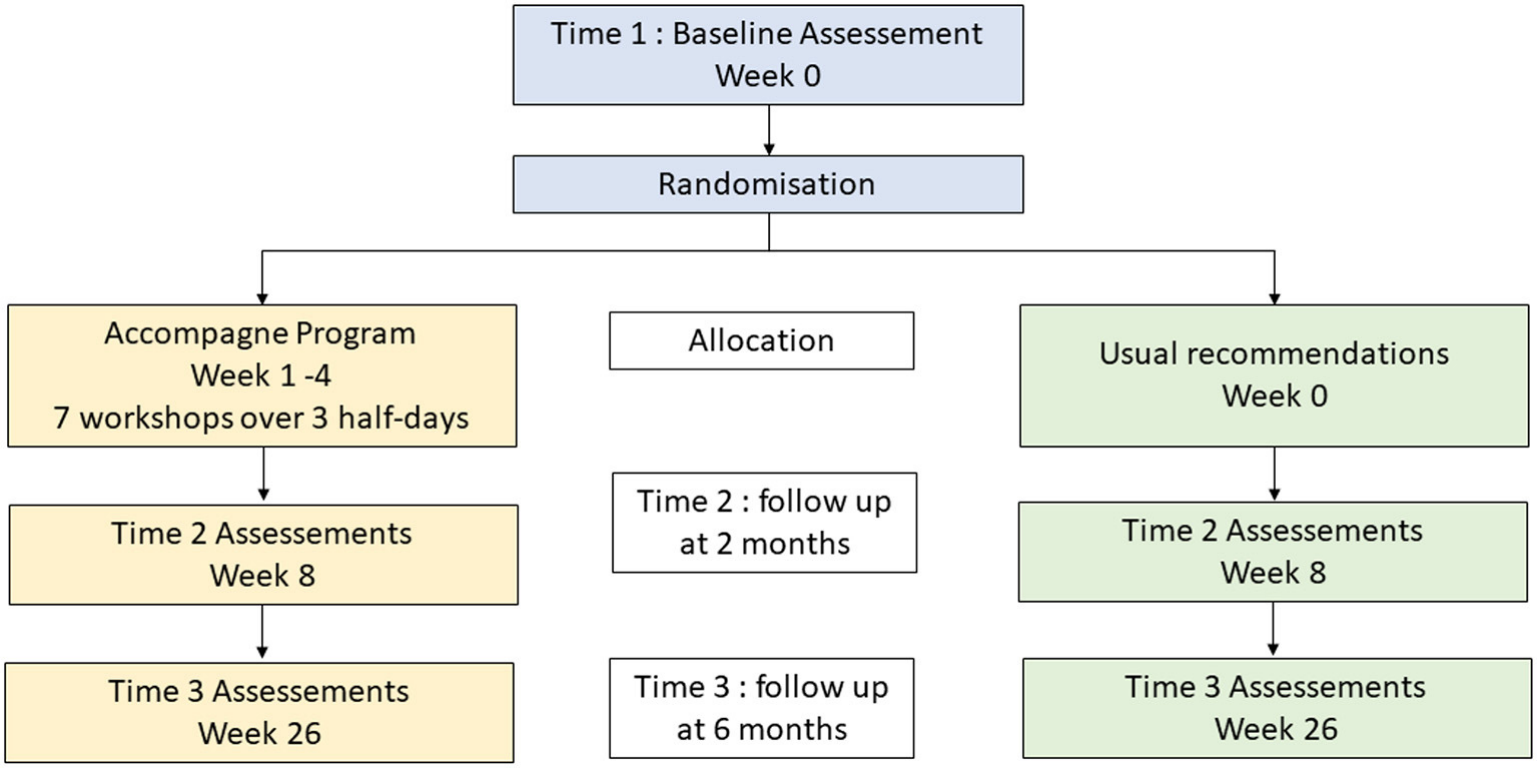
Abridged CONSORT diagram.

There will be three assessments: a baseline assessment (Time 1), another 2 months after baseline (Time 2) and a third 6 months after baseline (Time 3), as shown in Figure 2. Participants will be randomized between two sequences (i) Baseline (Time 1)>Intervention>Time 2>Time 3 or (ii) Baseline (Time 1) >Control>Time 2>Time 3 (as shown in Figure 2). The intervention will be based on seven collective workshops, spread over three half-days (once a week for three consecutive weeks). For participants recruited by Lyon Hospital, the evaluation will also include a driving simulator test at Baseline (Time 1), Time 2 and Time 3.

### Intervention description

#### Experimental group

Dyads included in the ACCOMPAGNE educational program group will take part in seven collective workshops, spread over three half-days (once a week for three consecutive weeks). Each workshop lasts approximately an hour and a half and uses educational content and pedagogical methods. Materials and methods of each workshop are described in Table 1.

**Table 1:** ACCOMPAGNE program workshops.

**Workshop (duration)**	**Main objective and methods**	**Materials (example)**
Workshop introduction (1 h)	Main objective: To increase participant's involvement by considering him/her as a key member of the workshops and to initiate a group dynamic.	
	Method: Round table: each participant answers the questions on the “question wheel”: Name, first name, age, place of residence. Do you like to drive? Do you drive often? For which activities? Do you experience any difficulties when driving? Motor skills? Concentration? Memory problems?	
Cognitive skills workshop (1h30)	Main objective: to facilitate awareness of the cognitive skills involved in driving (visuo-spatial, attentional, memory, executive).	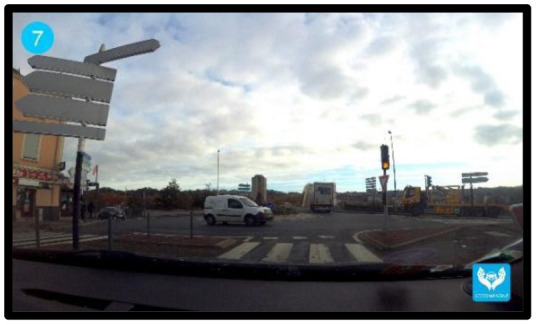
	Method: After defining the different cognitive mechanisms involved in driving, role plays are distributed. The participants have to imagine the cognitive mechanisms involved in each situation.	
Perception and environment workshop (1h30)	Main objective: To facilitate awareness of sensorimotor skills required to drive safely.	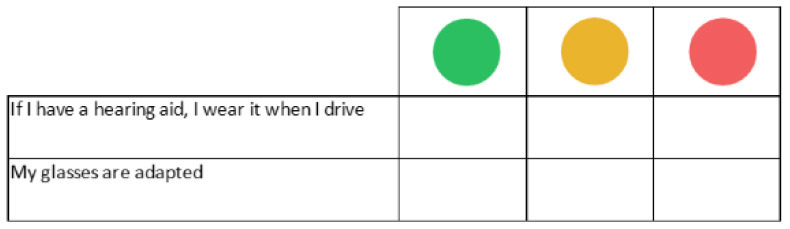
	Method: For each visual skill (acuity, accommodation, contrast and distance vision) the facilitator creates a discussion around the questions “What role does this skill play in driving? What is the risk if this skill is impaired?” Establishment of a checklist based on sensory status and level of alertness green light = safe driving/orange = adaptation required/red = do not drive	
Driving responsibilities workshop (1 h30)	Main objective: To facilitate awareness of accident risks linked to a driving affected by cognitive disorders or illness. To facilitate awareness of one's own responsibilities as a driver.	“Mrs. A., 82 years old, attends memory clinic for cognitive disorders and attentional difficulties. She also has a cataract. She uses her car several times a week to do her shopping. On this particular day, it is raining. Mrs A has to turn right, she does not see the bicycle that was riding behind her and hits it. The cyclist is injured and apparently has a broken arm. The cyclist is taken to hospital by the fire brigade and will probably need an operation.” What do you think are the administrative steps to be taken for Mrs A.?
	Method: Reflection on situation vignettes. What are the responsibilities? Does she need to have her driving assessed? In the long term, what steps can she take to assess her driving?	
Patient workshop (1 h)	Main objective: To share personal experience with driving and with driving adjustments because of cognitive or sensory disorders.	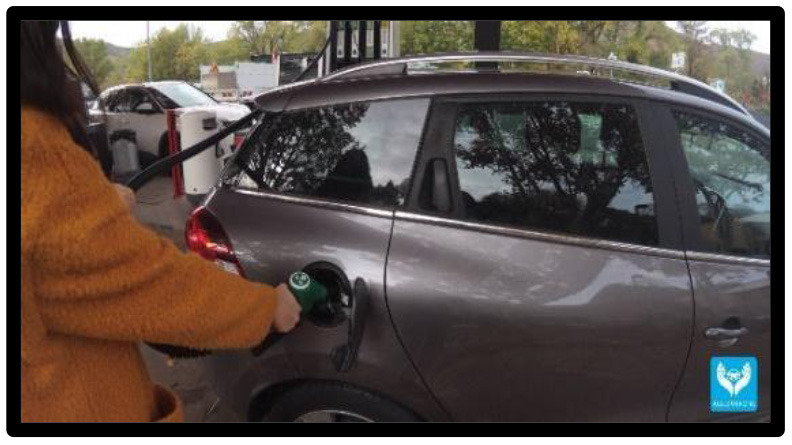
	Method: Patients are asked to write 8 words or short sentences on 8 post-its (one idea per post-it) around the question “What are the abilities needed to drive safely? A collective meta-plan is made to synthesize all the ideas. Each patient then chooses a photo. The psychologist allows the patients to discuss their representations, and relies on the sharing of experiences to soften the impact of giving up driving.	
Caregiver workshop (1 h) (at the same time than representation workshop)	Main objective: To allow speaking time to the caregivers so they can ask the questions they cannot ask when their relative is present.	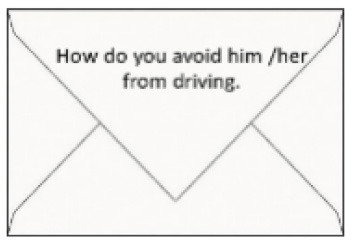
	Method: Envelope method. The natural caregivers ask their questions freely. The facilitators write down each question on a different envelope. Each participant will have to provide solutions to the questions in each envelope in turn (based on their own experience or on information acquired in the previous workshops). A collective discussion then allows the proposed solutions and possible adaptations to be listed.	
Driving strategies and alternatives workshop	Main objective: To establish required strategies or means for safe and autonomous driving.	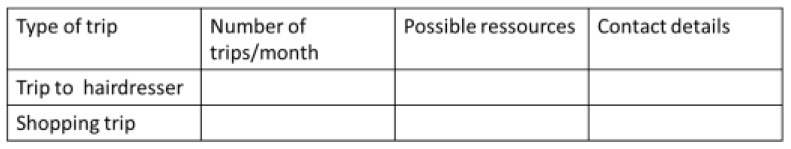
	Method: Presentation of risky situations (Night driving, rush hour, city traffic, unfamiliar or long routes, intersections, roundabouts, bends, insertions, difficult weather conditions, motorways, telephones, radio, chatter, physical pain, drunkenness, emotional stress..) and group discussion on possible solutions or alternatives (avoidance, adaptations like turn off the radio in challenging situations, planning the journey ahead of time…). Brainstorming about resources that can be used to avoid some driving situations and making a dyad specific alternative transportation plan.	

A neuropsychologist begins by introducing the program and explains the educational objectives in the *Introductory workshop*.

The neuropsychologist then proposes the *Cognitive skills workshop*. The objective is to help participants and their caregivers to understand the main cognitive functions and behavioral skills (self-control, stress management, compliance with instructions) involved in driving activity. The material provided for this workshop consists of photographs showing several driving situations which are known to be complex for older individuals (heavy traffic, unexpected events, dangerous intersections). The material will help individuals to think about the cognitive functions involved in driving activity, and to better understand the impact of these functions on driving activity. Participants are invited to think about the consequences of cognitive disorders in each of these situations. At the end of the workshop, participants ask any questions they might have, and explain their position regarding the continuation of driving activity.

Next, a physician or a nurse introduces the *Perception and environment workshop*. The objective is to help participants to be aware of the sensorimotor skills needed for safe driving (visual, auditory, motor, gestural skills, proprioception). For each skill (visual acuity for distance and near visual field sensitivity to glare, contrast vision, auditory skills, balance and proprioception functions, motor skills and gestures), participant groups determine the impact of aging and of the main age-related diseases. In this workshop, the effects of medication on driving are discussed, as well as anything else that can impact alertness at the wheel (fatigability, drowsiness). Participants are asked to classify each skill based on their personal situations as either green lights (no risk identified), vigilance points, or red lights (identified danger), and to list the possible actions needed to avoid any risks related to impaired skills (for example, avoiding driving at night).

In the following stage the physician presents the *Responsibilities workshop*. The objective is to help participants to put themselves in the position of a responsible driver, and to fully understand the responsibilities of everyone involved in a given situation. A brainstorming method is used. Stories are presented. These feature characters in driving situations in which their responsibility is involved. Participants are then asked questions. A number of legal aspects are discussed, particularly those related to medical conditions which result in an inability to drive. The procedure for responding to a certified physician, the responsibilities of each person, the legal obligations of health professionals and personal obligations relating to the driver and the vehicle are all described and detailed. The procedures used to assess their fitness to drive are also presented.

A psychologist then goes on to present the *Patient workshop*. The objective is to provide a listening place for participants only. Representations, fears, projections related to driving and cessation are discussed. The photo language method is used. Participants are invited to express the value they place on driving, and their feelings about a possible cessation.

At the same time another psychologist presents the *Caregiver workshop*. The objective is to provide a listening place for caregivers only. Questions related to the procedure involved in the fitness to drive assessment, to anosognosia or defensive positions, and to their relative's potential opposition to giving up driving are all discussed. This workshop also presents the attitudes that should be adopted and the adaptive measures that can be implemented.

An occupational therapist or a nurse holds a *Driving strategies and alternatives workshop*. Risky driving situations will be presented to the participants and they will discuss, in the group, about possible solutions or alternatives to such situations (avoidance but also other adaptive strategies like turn off the radio in challenging situations, planning journeys ahead of time…). Participants identify which trips are essential in their daily life, and decide which travel mode corresponds best to their needs and capacities. Various strategic or tactical adaptation behaviors for safer driving are discussed with the participants, such as the use of family, friends, associative and municipal resources, alternative transport options and strategies for avoiding risky situations. This workshop will be adapted to each dyad with a list of alternatives depending on the dyad's living place.

The interventions will be standardized, and each center will be provided with a kit containing an educational guide with the specific objectives of each workshop, the key messages, the facilitation techniques to be used, with detailed instructions, the duration of each activity in each workshop and the tools needed during the workshop. An explanatory workshop will take place for all participating health-care professionals before the start of the study. Moreover, to be involved in the workshop, professionals must be trained as a group facilitator and must have at least 3 years' experience in geriatric units.

#### Control group

participants and caregivers included in the non-experimental group will receive the conventional recommendations for driving a car. These will be provided by their physician during a consultation in the memory clinic, either when they are given their diagnosis or during a follow-up visit. These recommendations consist of a description of the participant's cognitive and sensorimotor risk factors for driving, advice on ways to adapt driving, or a recommendation to stop driving. Information about the procedure involved in responding to a certified physician is also provided.

### Outcomes and assessment tools

#### Main outcome: Implementation of self-regulation strategies

The main outcome is the implementation of self-regulation strategies in driving. Self-regulation strategies will be measured by the participants' scores on the DPPQ “Current Self-Regulatory Practices” subscale (29). The 2-month and 6-month scores of the control group and the experimental group will be compared. The subscale includes eight questions about the frequency of drivers' self-regulation strategies. These strategies consist of: waiting for the rain to stop before driving; asking someone to accompany them rather than driving alone; looking for parking lots to avoid parallel parking; avoiding turning left in traffic; avoiding taking the freeway; avoiding rush-hour traffic; avoiding driving in crowded places; and avoiding driving at night. A four-point scale (0: never, 1: rarely, 2: sometimes, 3: often) reflects the frequency of each item, creating a total score ranging from 0 (never uses any of these strategies) to 24 (uses all strategies often).

#### Secondary outcomes

-Driving changes:

The secondary objectives focus on driving changes observed by the participants themselves, and changes in the participant observed by the caregiver (see Table 2). Several indicators will be used to obtain these measurements.

**Table 2:** Measurements at baseline, 2 months and 6 months after the intervention on the patient and his caregiver.

**Outcome**	**Measure**	**Filled out by the:**		
		**Patient**	**Caregiver about the patient**	**Caregiver about himself**
Implementation of self-regulation strategies in driving	DPPQ's “Current Self-Regulatory Practices” (29)	x		
Indicators of driving changes	DHQ (30)	x		
Indicators of changes perceived by the caregiver	DHQ and DPPQ scales		x	
Indicators of driving ability self-awareness	Second part of the DHQ scale, objective measures from driving simulator and subjective measures from a questionnaire administered just after each simulator task.	x		
Indicators of mood effects of driving modifications	GDS (39)	x		x
Indicators of quality of life effect of driving modifications	QoL-AD scale (40)	x		x
Indicators of caregiver burden of driving modifications	ZBI (41)			x

Indicators of driving changes in participants will be measured at 2 months and 6 months by a composite score calculated using the first part of the Driving Habits Questionnaire (DHQ) (30) and the other DPPQ sub-scales. Indicators of driving changes perceived by the natural caregiver will be measured at 2 months and 6 months by a composite score which includes scores from the DPPQ and DHQ scales, and scores calculated from observations made by the caregivers about participants' driving. Indicators of self-awareness of driving ability will be measured at 2 months and 6 months by the score obtained in the second part of the DHQ scale filled out by the participant. Those questionnaires (DPPQ and DHQ scale) are self-reported measures but since the patient and the caregiver both answer about the patient driving, we may control for most of the bias induced by such measures. Moreover, authors such as Charlton et al. showed that results with objective methods about self-regulation of driving are coherent with self reported measures (24) which supports our use of those questionnaires.

-Driving performance and self-awareness of driving ability:

Driving performance and indicators of self-awareness of driving ability will be measured for participants who perform tasks on the driving simulator. Driving performance will be evaluated by 6 driving tasks, each of which will represent one scenario: (1) A speed maintenance task to measure the ability to respect a speed limit of 80 km/h and to maintain lane position; (2) A car-following task to measure the ability to keep the vehicle in lane while maintaining a safe distance; (3) an overtaking task to assess the ability to make correct decisions when overtaking a vehicle when driving on a high-speed road; (4) Driving in a rural area to measure the capacity to adapt to different road situations such as stop signs; (5) Driving in an urban area to evaluate the ability to adapt when driving in town, i.e. responding to traffic lights and adapting to unexpected events such as pedestrians crossing, a vehicle pulling out of a parking space suddenly; (6) A braking task to measure the ability to brake quickly. These tasks were designed to assess driving performance in different situations varying in difficulty. That is why we used a variety of driving situations often encountered while driving: high-speed road, rural area and an urban area. Moreover, the variety of the tasks [following a vehicle (1), respect speed limit and maintain lane position (2), overtaking (3), adapt to different road situations and unexpected events (4, 5) and braking (6)] assess different abilities (e.g., motor, attentional) required for driving and are frequently used in driving simulator studies (42–44). To avoid fatigue effects, the driving tasks are short (no longer than 5 min).

For each task, means and standard deviations of speed, lane position, steering angle, reaction times to traffic lights or unexpected events will be measured. In addition to the objective measures obtained from simulator data, subjective measures will be collected from a questionnaire administered to the participant immediately after each simulator task. While the participant will perform the driving task, the experimenter will fill out the same questionnaire about participant's driving ability (i.e., perception of participant's driving ability). The comparison between the scores obtained from the participant and the experimenter will inform on the participant's driving ability self-awareness. Finally, objective as well as subjective measures will inform on the participant's driving ability and his/her self-awareness of this ability, respectively.

- Mood:

Indicators of mood effects on driving modifications will be measured by the Geriatric Depression Scale 15-item version (GDS 15) (39) in both participants and their natural caregivers at 2 and 6 months.

- Quality of life:

Indicators of quality of life following driving changes will be measured by the Quality of Life in Alzheimer's Disease Scale (QoL-AD) (40) in both participants and their natural caregivers at 2 and 6 months.

-Caregiver burden:

Indicators of caregiver burden following driving changes by the participant will be measured 2 and 6 months by the Zarit Burden Inventory (ZBI) (41) for natural caregivers.

### Sample size and data analyses

The self-regulation strategy in driving will be considered beneficial if the score of “Self-Regulatory Practice” increases on average by two points (24) 2 months from inclusion. The number of subjects was computed by considering a Student *t*-test for equal variances between the two groups, with a two-sided alpha risk of 5% and a power of 80%. Based on the sample size calculation, 90 participants per group is considered sufficient to detect differences between the two assessments. Because a 10% loss of dyads is expected during the follow-up period, 100 participants per group will have to be recruited, i.e. a total of 200 participants.

Kolmogorov-Smirnov tests will be used to determine the normality of variables. For demographic, clinical, neuropsychological variables, and scores obtained from scales (e.g., the DPPQ), between-group differences will be examined using Fisher's Exact tests, independent Student *t*-tests or Wilcoxon rank-sum tests, as appropriate. The effect size in the intervention group compared to the control group will be quantified by estimating the difference in mean scores between the 2 groups (experimental vs. control) with a 95% confidence interval. A multiple linear regression model will be performed with DPPQ “Current Self-Regulatory Practices” as the dependent variable, and the level of cognitive impairment at inclusion and other characteristics that may have an effect on the outcome criterion as factors. A mixed-effect linear regression model will also be computed with DHQ, GDS-15, Qol-AD and ZBI as dependents variables, and the group (experimental vs. control) and follow-up time (inclusion, 2 and 6 months) as independent variables. The interaction between the intervention group and the follow-up time will be observed.

The concordance between the performance on the driving simulator and the perception of driving ability measured immediately after simulated driving will be estimated by the Lin concordance correlation coefficient and by the Bland & Altman method for continuous criteria, and by the Cohen Kappa coefficient for ordinal criteria. The number of participants who significantly change their estimation at 2 and 6 months compared to before the intervention will be measured from objective and subjective measures at the different points in time.

### Procedure

Participants who are more likely to participate in the study will be identified during attendance at the memory clinics or in the geriatric departments of the four centers. The inclusion criteria will be checked during the pre-inclusion visit. During the visit to determine inclusion, a physician will take the medical history and note any comorbidities (including a diagnosis of cognitive disorder). He/she will also perform a sensory-motor examination using the Short Physical Performance Battery (balance, walking speed, chair raising), the Stop Walking when Talking (motor and verbal double task) and the Handgrip strength to detect physical frailty (Fried's criteria). Socio-demographic data and current medication will also be noted. A nurse will perform a visual examination by assessing the uni and binocular distance visual acuity (Monoyer scale) and near visual acuity (Parinaud scale). He/she will also perform a screening for age-related macular degeneration (Amsler grid), explore the visual field (with a finger) and examine the color perception. A neurocognitive examination will be performed by a neuropsychologist using the Victoria Stroop Test, the Trail Making Test (A and B), verbal fluencies (“P” and “Animals” in 2 min), the Rey Figure copy, and Digit Span and Coding from the WAIS-IV.

On the day of the inclusion visit, the neuropsychologist will administer the DPPQ, DHQ, QoL-AD, GDS-15 scales to the participant. Natural caregivers will respond by themselves to the DPPQ and DHQ scales (based on observations made about the participant's behavior), and to QoL-AD, GDS-15, ZBI. The dyads included in the control group will receive the usual medical recommendations on the same day. The dyads included in the intervention group will be invited to the three half-day workshops. Workshops will start as soon as the experimental group contains four dyads. At 2 and 6 months, the neuropsychologist will administer the DPPQ, DHQ, QoL-AD, GDS-15 scales to the participant. Caregivers will complete the DPPQ and DHQ scales, (based on observations regarding the participant's behavior), and the QoL-AD, GDS-15, ZBI on their own. A questionnaire about life events that took place between each visit will also be administered to the patient and the caregiver at 2 and 6 months. For 40 participants recruited in the Lyon center, observations on a driving simulator will be collected at inclusion, at the 2-month visit and at the 6-month visit.

### Potential pitfalls and unintended effects

This study may face different difficulties. As it tackles the delicate question of driving among olders and more specifically among olders with neurocognitive disorders, we could face difficulties in recruiting participants. Indeed, it is possible that participants with neurocognitive disorders may not take part in this study due to a fear of license loss, even if there is no implication for reporting medically at-risk drivers to the jurisdiction's governing authority. Moreover, we need to include patient/caregiver dyads, thus, in order to be involved in the study, the patient will need to have a natural caregiver, available and willing to participate which reduces the number of potential participants. If recruitment difficulties turn out to be too important, it could lead to a small sample size, which may limit the generalisability of the results. In addition, uncontrolled intercurrent variables related to the individual history of the disease could bias our result. That is why, we choose to control for such variables as best as possible by using questionnaires about life events at 2 and 6 months. Furthermore, the intervention duration (i.e., 3 weeks) may not be sufficient to produce the expected outcomes. Lastly, for the subgroup of patients who undergo a driving task, the use of the driving simulator can generate simulator sickness and may frighten or shock the patient if he/she makes a serious mistake in the driving scenarios such as hitting a pedestrian or another car.

## Discussion/conclusion

For older people with cognitive disorders, driving cessation can lead to a series of negative changes in terms of autonomy and mood for both the patient and the natural caregiver. Recent studies agree on the need to implement interventions to provide support for patients and natural caregivers during the process of adaptation, and then on cessation of driving (14, 15). However, the studies carried out so far have focused on targeted interventions for patients only or natural caregivers only (16, 19, 20), even though interactions between patient and caregiver seem to be at the heart of this process. The onset of cognitive impairment requires the adaptation of driving activity, rather than its abrupt cessation. An intervention program which makes both the patient and the natural caregiver actors in the process of giving up driving seems therefore necessary. This should cover all aspects involved, beginning with an awareness of driving ability, and the subsequent implementation of self-regulated driving strategies, all the way through to complete cessation of driving. This cognitive, social and psychological support should also help to maintain the quality of life and mood of patients and their natural caregivers despite driving limitations. It is also important to consider the environment in which the patient lives. Travel needs and dependence on a car are not the same depending on where the patient lives (e.g., in the countryside or in the city). Severity of disease and the associated road risks will also have to be estimated in any analysis of the maintenance or cessation of driving for these patients. The results will provide information on how to optimize the care of people suffering from neurocognitive pathologies. If the results are conclusive, this approach could be extended to all other centers dealing with this problem.

## Ethics statement

The Regional Ethics Committee (Comité de protection des personnes SUD-OUEST ET OUTRE-MER II) has approved the study (Decision reference number: 20.01612.220050). Authorization for handling these data was granted by the French Data Protection Authority (CNIL: Commission Nationale de l'Informatique et Liberés). The practitioner will give an information document explaining the objectives and the protocol of the study to the patient and the natural caregiver. They will have enough time to think about their participation in the study. Informed written consent will be obtained from all participants before the start of the study.

## Author contributions

FD-C and M-HC initial idea, drafted, revised, and approved the protocol and manuscript. RB, MG, and ML drafted, revised, and approved the protocol and manuscript. LP-F and MR initial idea and revised and approved the protocol focusing driving abilities. CG and PK-S drafted, revised, and approved the protocol and manuscript and obtained funding. All authors have made substantial contributions to conception and design, acquisition of data, and read and approved the final manuscript.

## Funding

This study was supported by a grant from the Direction Générale de l'Offre de Soins (DGOS-PHRIP; grant number: 19-0034) and by the Délégation de la Sécurité Routière.

## Conflict of interest

The authors declare that the research was conducted in the absence of any commercial or financial relationships that could be construed as a potential conflict of interest.

## Publisher's note

All claims expressed in this article are solely those of the authors and do not necessarily represent those of their affiliated organizations, or those of the publisher, the editors and the reviewers. Any product that may be evaluated in this article, or claim that may be made by its manufacturer, is not guaranteed or endorsed by the publisher.
